# How I do it: Simple and effortless approach to identify thoracodorsal nerve on axillary clearance procedure

**DOI:** 10.3332/ecancer.2012.255

**Published:** 2012-05-28

**Authors:** T Zin, M Maw, SM Oo, DR Pai, RB Paijan, M Kyi

**Affiliations:** 1 Department of Surgery, Melaka Manipal Medical College, Melaka, Malaysia; 2 Department of Surgery, Hospital Pakar Sultanah Fatimah, Muar, Johor, Malaysia

**Keywords:** *thoracodorsal nerve*, *axilla*, *axillary clearance*

## Abstract

Breast cancer surgery frequently involves an axillary clearance procedure for nodal metastases. Injury to the thoracodorsal nerve is one of the complications related to the axillary dissection. The thoracodorsal nerve innervates the latissimus dorsi muscle which facilitates in certain movements of the arm. Moreover, it can be used as a nerve graft in long thoracic nerve injury whether in trauma or surgery. Understanding the anatomy structures and good surgical technique in the axillary clearance procedure can identify and prevent such an injury to the thoracodorsal nerve. Here, we demonstrate a simple and effortless technique for identification of the thoracodorsal nerve during axillary surgery.

## List of abbreviations

TDN: thoracodorsal nerve

## Introduction

Axillary clearance is one of the most common surgical options for breast-cancer patients with axillary nodal metastases. It is generally defined as clearance of the axillary contents; fat and lymph nodes positioned at the axillary space where axillary vein serves as the superior boundary. Thoracodorsal nerve (TDN) is one of the nerves that need to be identified and preserved during the axillary clearance procedure. It contributes pure motor innervations to the latissimus dorsi muscle for extension, rotation, and adduction of the arm. Moreover, TDN can be used as a nerve graft for patients having long thoracic nerve (C5, C6, C7) injury in trauma [1] and operations. Because of the above-mentioned important reasons, identification of TDN and its anatomical relationship with other anatomical structures is crucial during the axillary clearance procedure. In 2001, Vijey et al. mentioned one of the easy methods to identify TDN during axillary dissection where the nerve was identified first [2]. In this article, we would like to share our experiences about how we dissect and identify the TDN simply and effectively during the axillary clearance procedure.

## Clinical anatomy

The original nerve fibres of TDN emerge mainly from C7, C8, and some nerve fibres from C6, and they ultimately appear as one of the branches of the brachial plexus posterior cord. Its size is about 2 to 3 mm during its axillary course. TDN runs underneath the axillary vein during the descend into the axilla and ends up inside the latissimus dorsi muscle for motor innervations. The voyage passes behind the axillary vein, and makes its first appearance in the axilla just underneath the lateral thoracic vein: one of the consistent branches from the axillary vein. After that point, TDN changes direction downwards and laterally to join with subscapular vessels and forms thoracodorsal neurovascular bundle, which innervates the latissimus dorsi muscle. TDN usually lies superficially to the thoracodorsal vessel during its course inside the bundle. Another landmark that can constantly be visualized while tracking the TDN is a triangle formed by the TDN, axillary vein, and subscapular vessel located at the medial, superior, and lateral positions, respectively. These landmarks are constantly well seen in all our cases during axillary dissection. However, there is an aberrant course of the TDN in some reported series, which is not detected in our series [3].

## Technique

**Figure 1:**
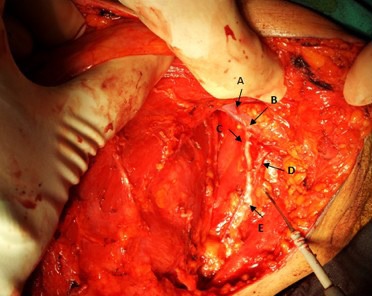
Operative view of left axillary clearance procedure showing axillary vein (A), cut end of lateral thoracic vein (B), thoracodorsal nerve (C), subscapular vein (D) and thoracodorsal neurovascular bundle (E).

**Figure 2:**
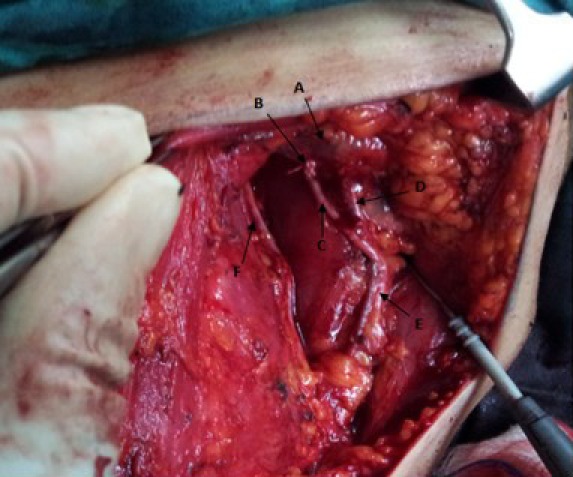
Operative view of left axillary clearance procedure showing axillary vein (A), cut end of the lateral thoracic vein (B), thoracodorsal nerve (C), subscapular vein (D), thoracodorsal neurovascular bundle (E) and long thoracic nerve (F).

During the axillary clearance procedure, the first dissection is to remove fatty tissue from the very apex of the axilla around the axillary vein. The axillary vein can be visualized simply as a bluish cord while dissecting yellowish fat globules around it after opening the fibrous fascia. The axillary vein is then dissected downwards and laterally along its course towards the arm. The lateral thoracic vein appears at the antero-inferior aspect of the axillary vein about 2 to 3 cm lateral to the chest wall. This is the landmark vein for the TDN. The TDN constantly lies just underneath the lateral thoracic vein. Careful dissection and ligation of the lateral thoracic vein exposes the TDN simply and effortlessly (Figures 1 and 2). The size of the nerve is usually around 2 to 3 mm and invariably well seen from the pinkish red subscapularis muscle background. Further dissection exposes the nerve, which travels infero-laterally for a short distance of about 3 cm and joins with subscapular vessels to form the thoracodorsal neurovascular bundle. The remaining distal part of the nerve lies inside the thoracodorsal neurovascular bundle and can be easily traced out during the remaining part of the procedure.
